# An analysis on intersectional collaboration on non-communicable chronic disease prevention and control in China: a cross-sectional survey on main officials of community health service institutions

**DOI:** 10.1186/s12913-017-2654-9

**Published:** 2017-11-10

**Authors:** Xing-ming Li, Alon Rasooly, Bo Peng, JianWang, Shu-yu Xiong

**Affiliations:** 1 School of Health Administration and Education, Capital Medical University, You An Men Wai Xitoutiao 10, Beijing, 100069 (P.N.) China; 2 Joyce and Irving School of Medicine, Ben-Gurion University of the Negev, Be’er Sheva, Israel; 3 School of Health Administration and Education, Capital Medical University, Beijing, China; 4 Department of Medicine, Peking Union Medical College, Chinese Academy of Medical Sciences, Beijing, China; 5 Department of Medicine, Peking Union Medical College, Chinese Academy of Medical Sciences, Beijing, China

**Keywords:** Integrated health care, Intersectional collaboration, NCD prevention and control, Community health, China

## Abstract

**Background:**

Our study aimed to design a tool of evaluating intersectional collaboration on Non-communicable Chronic Disease (NCD) prevention and control, and further to understand the current status of intersectional collaboration in community health service institutions of China.

**Methods:**

We surveyed 444 main officials of community health service institutions in Beijing, Tianjin, Hubei and Ningxia regions of China in 2014 by using a questionnaire. A model of collaboration measurement, including four relational dimensions of governance, shared goals and vision, formalization and internalization, was used to compare the scores of evaluation scale in NCD management procedures across community healthcare institutions and other ones. Reliability and validity of the evaluation tool on inter-organizational collaboration on NCD prevention and control were verified.

**Results:**

The test on tool evaluating inter-organizational collaboration in community NCD management revealed a good reliability and validity (Cronbach’s Alpha = 0.89,split-half reliability = 0.84, the variance contribution rate of an extracted principal component = 49.70%). The results of inter-organizational collaboration of different departments and management segments showed there were statistically significant differences in formalization dimension for physical examination (*p* = 0.01).There was statistically significant difference in governance dimension, formalization dimension and total score of the collaboration scale for health record sector (*p* = 0.01,0.00,0.00). Statistical differences were found in the formalization dimension for exercise and nutrition health education segment (*p* = 0.01). There were no statistically significant difference in formalization dimension of medication guidance for psychological consultation, medical referral service and rehabilitation guidance (all *p* > 0.05).

**Conclusion:**

The multi-department collaboration mechanism of NCD prevention and control has been rudimentarily established. Community management institutions and general hospitals are more active in participating in community NCD management with better collaboration score, whereas the CDC shows relatively poor collaboration in China.

Xing-ming Li and Alon Rasooly have the same contribution to the paper.

Xing-ming Li and Alon Rasooly listed as the same first author.

## Background

### The rising of NCD in China

In recent 40 years, China has gone through fast social and economical transformations, including industrialization, urbanization, and aging, which contribute to the rising number of Chinese people with Non-communicable Chronic Disease (NCD), and today the number of persons diagnosed with NCD is estimated at above 260 million [1, 2]. As the other developing countries, tobacco use, foods high in saturated and trans fats, salt, and sugar, physical inactivity, and the harmful consumption of alcohol all serve as the underlying causes of NCD [3]. These modifiable risk factors disproportionately affect individuals with NCD among lower socio-economic groups and further perpetuate health and economic inequalities [4].

### Background on health institutions against NCD in China

As part of the ongoing health reform, China issued a Multi-ministerial Plan for NCD Prevention and Control [2] in 2012. Describing and comparing the assigned roles and responsibilities of the different health institutions in treating NCD may provide more insights into China’s NCD management.

According to the document, General Hospitals (GH) shall:"(1) carry out registries for chronic disease related information, (2) provide diagnosis, treatment and rehabilitation services for patients with critical and acute NCD, (3) provide technical guidance to primary healthcare institutions for NCD diagnosis, treatment and rehabilitation,(4)also establish two-way referral mechanisms between the hospitals and primary health care institutions.".

As the primary health care institutions for NCD prevention and control run by governments, Community Health Service Institutions (CHSI) play an impoartant role in offering basic medical and public health services, which are regarded as the basic networks for medical treatment and public health to inhabitants of administrative area, and the main nonprofit primary institutions for NCD management, CHSI shall: "(1) perform regular monitoring and follow-ups on high-risk groups detected during physical examinations and screening, and implement targeted interventions and reduce morbidity risks, (2) strengthen management and services for patients with hypertension, diabetes, chronic obstructive pulmonary disease and other NCD, (3) provide follow-up and rehabilitation guidance for cancer patients,(4) be responsible for the execution and implementation of NCD prevention and control measures.".

The Center of Disease Control (CDC) of local community shall: "(1) develop and promote appropriate technologies to detect high-risk groups, strengthen lifestyle intervention, provide supervision and conduct evaluation, (2) set up specialized departments and designate personnel to conduct NCD prevention and treatment.".

Propaganda Authority(PA): as a unique health-associated agency in China, it is a government-run comprehensive communication department including radio, television, press and publication departments. Its role in the management of chronic diseases is to make full use of mass media to widely publicize the knowledge of NCD prevention and encourage people to consciously cultivate good habits and lifestyle.

Community Management Institutions(CMI): as an important partnership in communities, its responsibility includes organizing and motivating more people to attend the NCD prevention and promotion actions.

### Community-level integrated care for NCD management

The community is the junction of these departments and therefore an ideal place for NCD management. According to the World Bank Report (2011) on NCD in China, effective coordination between the above institutions and other service providers can guarantee the continuity of care needed to minimize the premature mortality and disability caused by ill-managed NCD. The services provided at different levels of the system should be enabled by well-defined NCD managing procedure, including early identification, active education and following-up, and referral procedures in community, based on successful multi-institution collaboration [5]. Examples of this approach from the North Karelia Project in Finland and from the Daqing Diabetes Prevention Study in China have shown that community level lifestyle modifications can significantly reduce morbidity from cardio-vascular diseases and diabetes, respectively [6, 7].

However, multi-departments collaboration of NCD prevention and control is difficult to evaluate, which is usually based on qualitative research methods, due to the lack of scientific methods and indicators. The research about collaboration content and structure is also insufficient, because the collaboration on community NCD management researches mostly focus on two-way referral [8–13]. Since NCD prevention and control is a multistep process involving particular procedures of management, special attention needs to be paid to how the health institutions collaborate in each procedure. Also as the main department for NCD prevention and control, how the community health service collaborates with other departments will affect the effect of NCD prevention and control.

Therefore, our study aims to provide evidence that may develop a measurement tool to evaluate the status of collaboration in NCD prevention and control procedures among the listed community health service institutions in China and ensure better collaboration effect on NCD prevention and control.

## Methods

### Participants and data collection

A stratified randomized sampling was used to selected the following several districts across China: Haidian and Fengtai Districts of Beijing, Tangu District of Tianjin, Wuchang District of Bubei and the city of Yinchuan of Ningxia, which were sampled respectively from each level of regional economic areas by simple randomized sampling method. Broadly speaking, Beijing and Tianjin are economically well developed, Wuhan moderately developed, and Ningxia developing. A computerized questionnaire was sent to the main officials of community health service organizations in the sampled regions accompanied by an official letter from each of the four city and provincial health departments stating the importance of the survey. Each returned questionnaire was carefully reviewed for its completeness and consistency. For those questionnaires with incomplete and/or inconsistent responses, one or two follow-up telephone calls were made to ensure completeness and consistency. The data from returned questionnaires were then transferred into a database for analysis. The investigation was carried out from October 2013 to May 2014. Altogether 444 questionnaires were sent out, and 444 ones were returned.

### Study design

Our study was conducted by a cross-sectional study, and its procedure includes the followings:

#### Demographic characteristics data collection

Demographic characteristics of the respondents including district, age, gender, professional title, specialty and work seniority in our survey were collected.

#### Development of evaluation frame of collaboration for NCD prevention and control between community health institutions and others

First, according to the working procedure previously described by Wen et al. [1], community NCD prevention and control work can be divided into the following procedures: physical examination, health record establishment, exercise and nutrition intervention, psychological consultation, medication guidance, medical referral service and rehabilitation guidance. Second, based on the stakeholder theory of NCD management, related institutions of NCD management were defined, including CHSI, GH, CDC, CMI and PA within the area.

To evaluate inter-organizational collaboration in the process of community NCD management in the sampling regions, a model and typology of collaboration between healthcare organizations validated by international studies was used [14, 15], which included four relational dimensions: governance, shared goals and vision, formalization and internalization, and ten indicators associated with these dimensions.

**Fig. 1:**
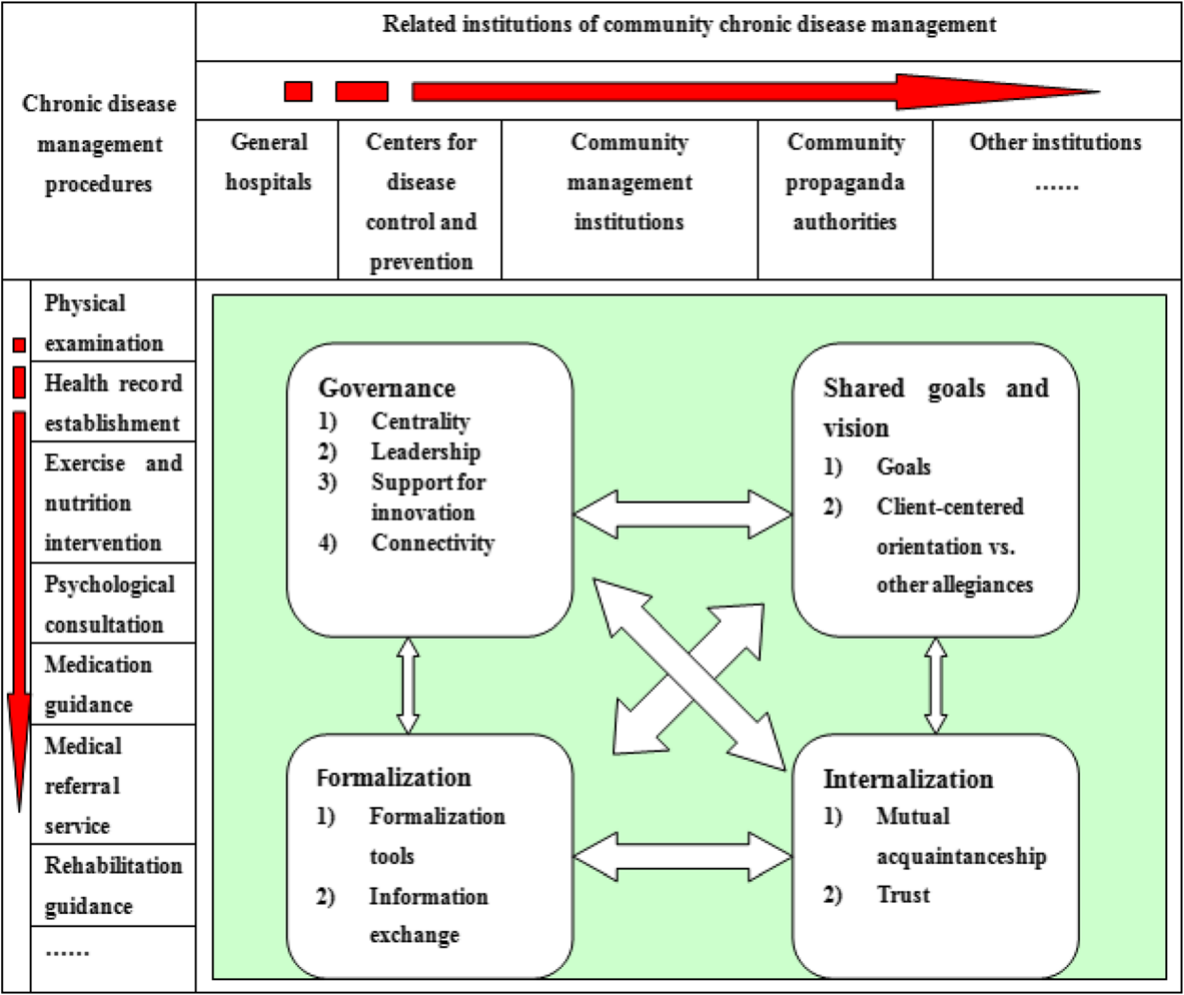
Evaluation frame of collaboration between community NCD prevention and control institutions

Based on literature analysis [14, 15], we constructed evaluative statements for the research participants to define the level of collaboration regarding each of the 10 indicators. We matched the level of collaboration with a Likert scale, from poor collaboration (equals a score of 1) to good collaboration (score of 3). Total points and averages of all the indicators were calculated to get the scores of different collaboration measures. In addition, we analyzed the collaborative status of different institutions during their NCD management procedures (see Fig. 1). The meaning of those ten indicators are presented as followings [14]: **Goals** is related to professional values in the form of common goals, with particular reference to the consensual and comprehensive nature of the goals; **Client-centred orientation vs. other allegiances** is refered to a complex structure of interests involving a variety of different types of allegiance: to the clientele, to the profession, to the organization, to private interests, etc.; **Mutual acquaintanceship** is refered that professionals must know each other personally and professionally if they are to develop a sense of belonging to a group and succeed in setting common objectives; **Trust for collaboration** is possible only when they have trust in each other’s competencies and ability to assume responsibilities; **Centrality** refers to the existence of clear and explicit direction that is meant to guide action; **Leadership** is necessary for the development of interprofessional and interorganizational collaboration. Leadership may take a variety of forms and can be categorized as either emergent or as related to a position; **Support for innovation** entails changes in clinical practices and in the sharing of responsibilities between partners. These changes represent real innovations that must be developed and implemented; **Connectivity** refers to the fact that individuals and institutions are interconnected, that there are places for discussion and for constructing bonds between them; **Formalization** is an important means of clarifying the various partners’ responsibilities and negotiating how responsibilities are shared; **Exchange of information** refers to the existence and appropriate use of an information infrastructure to allow for rapid and complete exchanges of information between professionals.Finally, we described and compared scores of the community NCD prevention and control collaboration scale between different segments and institutions.

### Statistical analyses

We managed database by using Epidata 2.0. All data were entered twice and checked. Statistical analysis was carried out using SPSS 20.0. Categorical variables were described in the form of frequency and percentage. Numerical variables were described in the form of mean ± SD based on the results of normal distribution test. The reliability of inter-organizational collaboration questionnaire was evaluated by calculating total scores and Cronbach’s Alpha of different dimensions and split-half reliability. The validity was examined by structural factor analysis to evaluate the construct validity of this scale [16]. *F*-test was used to compare the scores of evaluation scale of different procedures and departments in NCD management collaboration. All *p* values < 0.05 was considered statistical significance.

## Results

### Demographic information

This survey collected questionnaires from 444 main officials of community health service institutions, which covered 160 institutions in China. The majority of these institutions were from Beijing, accounting for 41.2%. Most of the respondents were from 40 to 49 years old, which accounted for 42.6%. Nearly 73% of the respondents were female, 44.0% who accounted for the highest proportion were healthcare workers with an intermediate professional title. As for specialty, the largest group of respondents specialized in general medicine with a total number of 162 and a proportion of 29.8%. Most respondents worked less than 10 years. See Table 1.

**Table 1:** Demographic information of 444 main officials of community health service institutions in China, 2014 [n (%)]

Category	Beijing	Tianjin	Wuhan	Ningxia	Total
Gender*					
Male	26(14.6)	18(39.1)	39(34.2)	34(35.1)	117(26.9)
Female	152(85.4)	28(60.9)	75(65.8)	63(64.9)	318(73.1)
Age (years)*					
≤ 30	12(6.7)	16(34.8)	13(11.2)	11(11.5)	52(11.9)
30~	55(30.7)	22(47.8)	31(26.7)	30(31.1)	138(31.6)
40~	83(46.4)	6(13.0)	50(43.1)	47(49.0)	186(42.6)
≥ 50	29(16.2)	2(4.3)	22(19.0)	8(8.3)	61(14.0)
Professional title					
Senior	14(7.7)	0(0)	6(5.3)	3(3.2)	23(5.3)
Deputy senior	57(31.5)	4(8.7)	13(11.4)	31(32.6)	105(24.1)
Intermediate	91(50.3)	8(17.4)	62(54.4)	31(32.6)	192(44.0)
Primary	17(9.4)	26(56.5)	30(26.3)	24(25.3)	97(22.2)
Undefined	2(1.1)	8(17.4)	3(2.6)	6(6.3)	19(4.4)
Specialty					
General Practitioner	100(46.9)	10(20.8)	25(17.7)	27(19.1)	162(29.8)
Hospital Specialist	38(17.8)	2(4.2)	30(21.3)	25(17.7)	95(17.5)
Prevention and healthcare	11(5.2)	11(22.9)	24(17.0)	36(25.5)	82(15.1)
Nursing	11(5.2)	4(8.3)	19(13.5)	18(12.8)	52(9.6)
Administration	45(21.1)	14(29.2)	27(19.1)	15(10.6)	101(18.6)
Medical technicians	1(0.4)	1(2.1)	5(3.5)	10(3.1)	17(3.1)
Others	7(3.3)	6(12.5)	11(7.8)	10(7.1)	34(6.3)
Work seniority (years)					
≤ 10	59(33.3)	35(76.1)	33(28.2)	46(46.9)	173(39.5)
10~	61(34.5)	6(13.0)	38(32.5)	24(24.5)	129(29.5)
20~	37(20.9)	3(6.5)	33(28.2)	22(22.4)	95(21.7)
≥ 30	20(11.3)	2(4.3)	13(11.1)	6(6.1)	41(9.4)

Note: ^*^represents data missing

### Reliability and validity of the evaluation tool of inter-organizational collaboration on NCD management

Reliability and validity of the questionnaire about inter-organizational collaboration in community NCD management revealed that: Cronbach’s Alpha for the whole investigation tool was 0.89. Cronbach’s Alpha for the formalization dimension was 0.80, and that of governance dimension was 0.81. On the other hand, Cronbach’s Alphas of shared goals and internalization were relatively lower. The split-half reliability was 0.84, which exceeded the recommended value of 0.70, thereby showed a fair reliability. Furthermore, structural factor analysis of validity test indicated that the variance contribution rate of an extracted principal component was 49.70%, which surpassed the recommended value of 30%, thereby revealed a relatively high reliability. Factor analysis showed the cumulative variance contribution rates of shared goals and vision, internalization, formalization and governance were 69.08%,65.84%,63.82% and 84.17% respectively; these all exceeded the recommended value, thereby showed good validity of these dimensions (Table 2).

**Table 2:** Reliability of the evaluation tool of inter-organizational collaboration in NCD management in China, 2014(n = 444)

Dimension	Number of terms	Cronbach’s Alpha	Split-half reliability	KMO	Bartlett	Variance contribution rate (%)
Shared goals and vision	2	0.452	0.452	0.50	0.000	69.08
Internalization	2	0.481	0.481	0.50	0.000	65.84
Formalization	4	0.803	0.754	0.75	0.000	63.82
Governance	2	0.812	0.812	0.50	0.000	84.17
Total scale	10	0.885	0.842	0.80	0.000	87.00

### Evaluation result of regional inter-organizational collaboration in each dimension

**Fig. 2:**
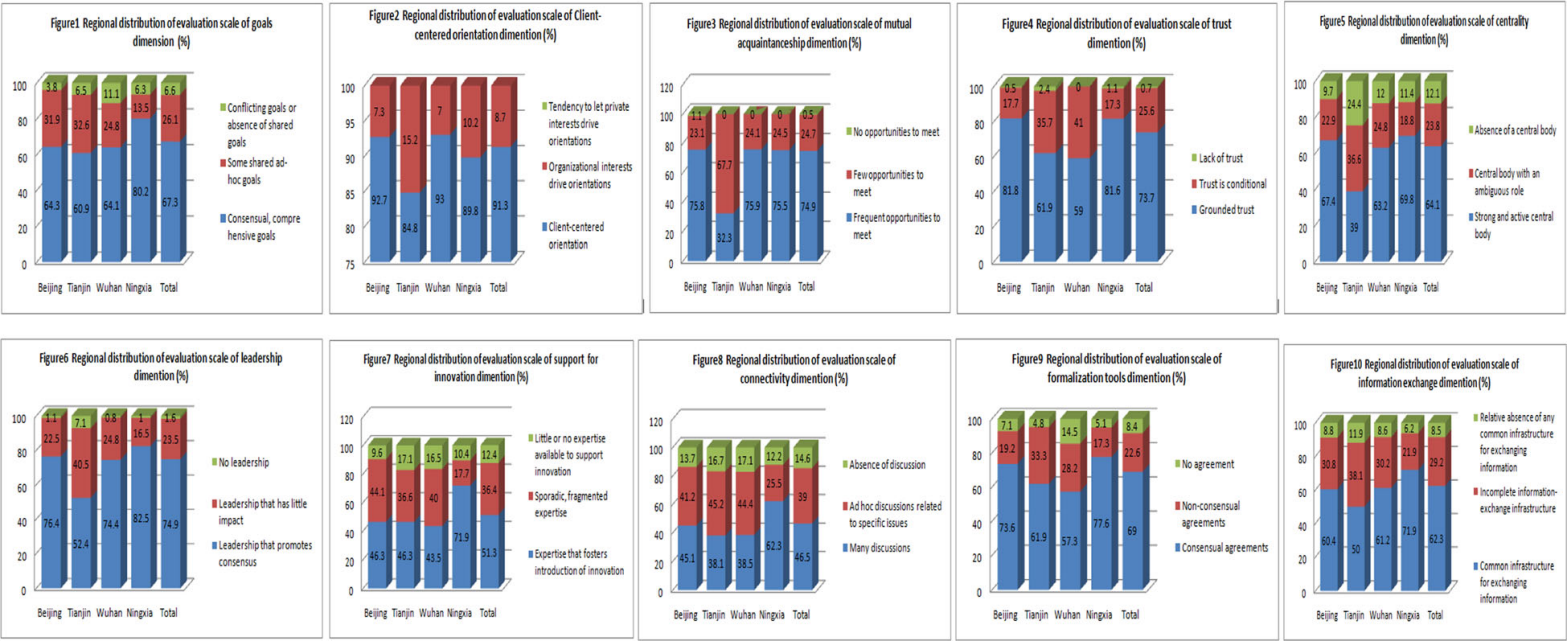
Regional distribution of evaluation scale of inter-organizational collaboration in NCD management in China, 2014 [*n* = 444, (%)]

Regional distribution of evaluation scale of inter-organizational collaboration showed that during NCD management in community, the score of shared goals, trust, centrality, leadership, support for innovation, connectivity and formalization tools dimensions showed significant differences for regional distribution (all *p* < 0.05),which indicated that regional factors exerted an influence on the mentioned collaboration contents (See Fig. 2).

### Evaluation result of inter-organizational collaboration of different institutions and management procedure in each dimension

With regard to the procedure of physical examination, the participation rate of CHSI was highest at 76%. Regarding formalization dimension, different departments showed significant difference (*p* = 0.01). Results showed that statistical difference existed among PA and GH, CDC, and CMI, indicating that PA had the highest score in the dimension of formalization. CMI had the lowest score, which suggested that due to professional differences, separate departments participated in physical examination collaboration to varying degrees.

With respect to the health record establishment procedure, the participation rate (76%) of CMI was the highest. In relation to governance dimension, formalization dimension and total score of the collaboration scale, their distribution had statistical difference (*p* = 0.01,0.00,0.00,respectively). As for governance dimension, further post-hoc comparison showed that the score of propaganda authorities was the highest, while that of CDC was the lowest. The same result was seen on the formalization dimension and total score of the collaboration scale. In other dimensions of health record establishment segment, no statistical differences existed between different intitutions (*p* > 0.05).

Regarding the exercise and nutrition health education segment, CMI and CDC showed highest participation rate at 66% and 61% respectively. In relation to formalization dimension, departmental differences were significant (*p* = 0.01). Further post-hoc comparison revealed that the score of PA was the highest, while that of CDC was the lowest.

Concerning the medication guidance procedure, the participation rate of CMI and GH were highest at 53% and 52% respectively. In relation to formalization dimension, departmental differences were significant (*p* = 0.04). Furthermore, post-hoc comparison revealed that the score of PA was the highest, while that of CDC was the lowest.

**Fig. 3:**
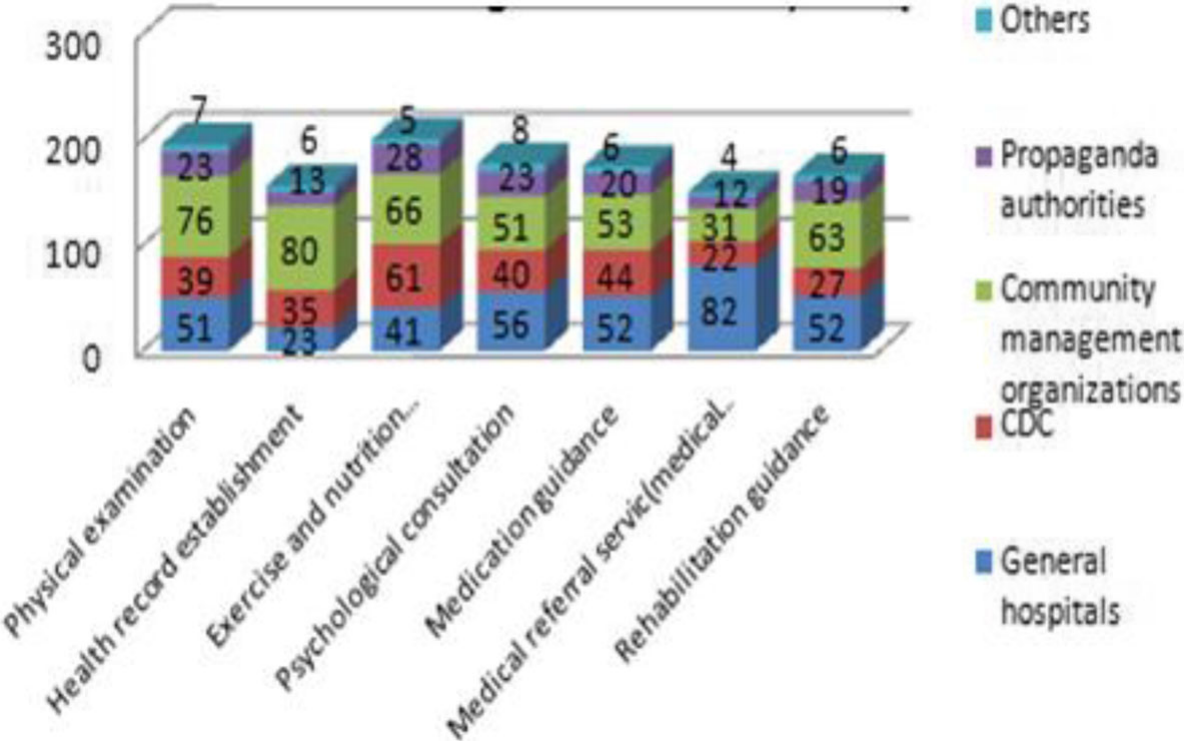
Participation rate of institution collaboration in NCD management in China, 2014

With regards to psychological consultation, both GH and CMI received high participation rates at 56% and 51% respectively. GH received the highest participation rates in segments of medical referral service (82%), being more than two times higher than that of CMI. Rehabilitation guidance participation rates were found to be highest at CMI at 63% and 52% at GH. Yet no statistical differences were seen between different institutions in each dimension of these segments (*p* > 0.05) (See Fig. 3 and Table 3).

**Table 3:** Departmental and segmental distribution of evaluation scale of inter-organizational collaboration on NCD management in China, 2014 [n = 444, n(%)]

Management procedures	Related institutions	Shared goals and vision		Internalization		Governance		Formalization		Total score of the collaboration scale	
		$\overline{x}$	s	$\overline{x}$	s	$\overline{x}$	s	$\overline{x}$	s	$\overline{x}$	s
Physical examination^1^	GH	5.48	0.79	5.54	0.69	10.08	2.01	5.17	1.16	26.26	3.59
	CDC	5.54	0.76	5.45	0.77	9.90	2.04	5.20	1.07	26.09	3.51
	CMI	5.56	0.75	5.42	0.78	9.84	2.18	5.10	1.24	25.91	3.89
	PA	5.61	0.71	5.52	0.74	10.42	1.99	5.58	0.95	27.13	3.17
	Others	5.64	0.68	5.54	0.79	10.21	2.20	5.50	0.96	26.89	3.52
*F*		0.67		0.93		1.57		3.54		2.23	
*p* value		0.61		0.45		0.18		0.01		0.06	
Health record establishment^2^	GH	5.49	0.72	5.48	0.70	10.13	1.88	5.28	0.99	26.39	3.20
	CDC	5.54	0.81	5.32	0.81	9.46	2.20	5.02	1.11	25.32	3.89
	CMI	5.53	0.78	5.46	0.74	9.98	2.10	5.17	1.20	26.14	3.75
	PA	5.72	0.63	5.49	0.77	10.70	1.82	5.72	0.59	27.63	2.65
	Others	5.50	0.71	5.61	0.78	10.39	2.25	5.72	0.57	27.22	3.32
*F*		0.80		1.24		3.23		4.74		3.88	
*p* value		0.52		0.29		0.01		0.00		0.00	
Exercise and nutrition intervention^3^	GH	5.46	0.79	5.52	0.72	10.07	1.89	5.22	1.05	26.27	3.40
	CDC	5.57	0.74	5.40	0.80	9.75	2.12	5.11	1.14	25.82	3.79
	CMI	5.54	0.75	5.47	0.74	9.96	2.20	5.12	1.25	26.10	3.83
	PA	5.52	0.75	5.49	0.80	10.46	1.93	5.51	0.95	26.98	3.33
	Others	5.50	0.79	5.61	0.78	10.06	2.21	5.61	0.85	26.78	3.39
*F*		0.47		0.83		2.17		3.31		1.99	
*p* value		0.76		0.50		0.07		0.01		0.09	
Psychological consultation	GH	5.51	0.75	5.59	0.67	10.28	1.84	5.28	1.03	26.66	3.22
	CDC	5.48	0.82	5.45	0.77	10.04	1.99	5.21	1.10	26.18	3.54
	CMI	5.49	0.81	5.51	0.74	10.14	2.13	5.26	1.15	26.40	3.81
	PA	5.49	0.79	5.58	0.79	10.63	1.84	5.63	0.84	27.34	3.11
	Others	5.41	0.80	5.59	0.85	9.91	2.43	5.45	1.06	26.36	3.92
*F*		0.10		0.70		1.16		1.95		1.28	
*p* value		0.98		0.59		0.33		0.10		0.28	
Medication guidance^4^	GH	5.49	0.77	5.52	0.71	10.06	1.96	5.18	1.16	26.24	3.51
	CDC	5.54	0.79	5.45	0.79	10.02	2.12	5.23	1.12	26.24	3.69
	CMI	5.51	0.80	5.51	0.75	10.15	2.06	5.26	1.11	26.43	3.58
	PA	5.58	0.76	5.58	0.79	10.58	1.89	5.67	0.88	27.42	3.11
	Others	5.44	0.81	5.50	0.89	9.94	2.29	5.56	0.89	26.44	3.67
*F*		0.21		0.31		0.84		2.43		1.26	
*p* value		0.93		0.87		0.50		0.04		0.29	
Medical referral servic(medical green channel)	GH	5.47	0.80	5.52	0.72	10.16	1.91	5.17	1.18	26.32	3.45
	CDC	5.53	0.80	5.47	0.75	10.25	2.02	5.25	1.06	26.51	3.53
	CMI	5.58	0.73	5.45	0.73	10.31	2.02	5.19	1.16	26.54	3.62
	PA	5.69	0.69	5.53	0.76	10.91	1.80	5.69	0.74	27.81	2.83
	Others	5.67	0.65	5.25	0.97	9.75	2.80	5.33	1.23	26.00	4.63
*F*		0.89		0.49		1.21		1.52		1.34	
*p* value		0.47		0.74		0.30		0.20		0.25	
Rehabilitation guidance	GH	5.40	0.81	5.50	0.73	10.29	1.99	5.19	1.20	26.39	3.66
	CDC	5.47	0.82	5.47	0.73	10.28	1.88	5.28	0.97	26.50	3.29
	CMI	5.54	0.76	5.51	0.68	10.16	2.00	5.09	1.25	26.31	3.63
	PA	5.65	0.66	5.61	0.70	10.75	1.92	5.49	1.03	27.49	3.16
	Others	5.50	0.73	5.44	0.89	10.06	2.57	5.38	0.81	26.38	3.96
*F*		1.15		0.34		0.89		1.34		1.16	
*p* value		0.33		0.85		0.47		0.25		0.33	

Note: General hospitals (GH),CDC, Community management institutions(CMI),Propaganda authorities(PA),Others. Analysis of variance and post-hoc comparison revealed that, 1: Difference existed between PA and GH, CDC, CMI; 2: In relation to governance dimension, difference existed between CDC and GH, CMI, PA; it was also seen between CMI and PA. On the formalization dimension, difference existed between PA and GH, CDC, CMI; it was also seen between other institutions and CDC, CMI. With respect to the total score of the collaboration scale, difference existed between CDC and GH, CMI; it was also seen between PA and CMI, others. 3: Statistical difference existed between PA and GH, CDC, CMI. 4: Statistical difference existed between PA and GH, CDC, CMI

## Discussion

Multi-institution collaboration of NCD management is an effective strategy to improve the effectiveness of NCD management in China. To yield long-lasting and comprehensive effects on health, efforts should be made on the basis of community, with the collaboration of the institutions of health, education, finance, environment protection and publicity [5–7, 17]. The Chinese government launched a new medical reform program from 2009, in which NCD management was considered as a basic and vital function for CHSI [2], who are encouraged to collaborate with multiple departments to prevent NCD, this collaboration has not yet been defined nor assessed by quantitative methods.

In this study, we firstly verify the reliability and validity of the multi-departments collaboration evaluation scale, which could provide us with a scientifical tool to measure the content of the current collaboration across institutions. Our survey suggested that among all collaboration dimension evaluation indicators (except connectivity dimension), the proportion of respondents choosing the highest degree of recognition reached more than 50%, which indicates that the NCD prevention and control collaboration mechanisms has been established in China [18]. However, the effect of the collaboration varies between different aspects or institutions. The combination of different institutions has not yet formed a combat force against NCD [19]. A substantial problem lies in the support given to innovation and information exchange: presently, collaboration uses old forms of meetings or projects, which need to be improved. For example, as for formalization score, multi-sector collaboration across physical examination, health record establishment, exercise and nutrition intervention and medication guidance showed significant difference.

The incompatibility in the collaboration is reflected in different procedures of NCD management. In health examination aspect, there was high participation of multi-departments in community management institutions, but those institutions scored low, while the propaganda institutions got the highest score, which indicates that the role of propaganda is very dominant, which could play an irreplaceable role in community organization and management of public affairs, mightily influencing all other departments. Other relevant studies also show that, to achieve the goal of community NCD management, we need to use the power of local partnership. A good relationship among various institutions indicated that, there was difference in participation between different departments, involved in the health examinations because of their professional levels [20], which suggests the need for strengthening the normative management in multi-departments collaboration for health examinations.

In the process of establishing health records, the CMI had the highest multi-department participation rate but the propaganda institutions had the highest management structure score, which showed that, most people gained their knowledge on NCD prevention through propaganda departments, covering a large range of the population, and delivering concise and convenient health educational information [21]. But the CDC is mainly in charge of the primary prevention in the prevention and control of NCD on the community levels, including organizing communities in promoting health, monitoring of NCD and relevant factors, surveillance and evaluation of the prevention and control of NCD, refinement and making of new intervention strategies and measures [22]. However, the CDC on each level showed poor capability in intervening the major NCD and relevant risk factors by collaborating with other departments, which showed that the CDC need better transformation from infectious disease prevention and control to NCD prevention and control, and they lack the capacity to coordinate with other related sectors [23].

In multiple links such as consulting, referral and rehabilitating service, the highest participation rate of multi-department was in the GH and CMI, which showed, the dominant role that GH played in the prevention and control of NCD cannot be changed, and further collaboration with community institutions should be improved. The CHSI is the main forum to realize the primary health care. In the course of NCD comprehensive management, however, the role of general hospital should not be undermined. The referral and rehabilitation procedure in NCD could be very professional and systematic, so that community institutions are confronted with problems such as the shortage of equipment and skillful physicians in China [24], which will influence the effect of collaboration between the GH and CHSI. Therefore, its comparison results showed no statistical difference, when compared to those between other relevant institutions and community hospitals in the management of NCD, which indicates a more detailed cooperative plan should be put forward to regulate the various departments’ responsibilities. It also calls for more effective measures to motivate more professional prevention and control institutions to join the community works. The above results further show that in China, even within health institutions, multi-department collaboration yielded few public health effectiveness on NCD prevention and control.

## Conclusions

In summary, our research developed a tool of evaluating intersectional collaboration on NCD prevention and control, by which we can find that the multi-department collaboration mechanism of NCD prevention and control has been rudimentarily established. CMI and GH are more active in participating in community NCD management with better collaboration effect, whereas the CDC shows relatively poor collaboration with other health institutions in China.

Based on the above status analysis, recommendation for policy makers will be put forward:

First, to further improve the awareness of the importance of collaboration of government institutions of various levels on the prevention and control of NCD; Second, to improve the governance structure of multi-sectoral collaboration in NCD management and strengthen the construction of the system and collaboration between the institutions of legal norms; Third, to further improve the government leading, institutions support and public participation in the collaboration mechanism; Fourth, to strengthen the information exchange and supervision and evaluation mechanism to ensure the smooth information communication.

## Abbreviations

CDC: Center for Disease Prevention and Control
CMI: Community Management Institutions
GH: General Hospitals
NCD: Non-communicable Chronic Disease
PA: Propaganda Authorities
